# Transplanted human cones incorporate into the retina and function in a murine cone degeneration model

**DOI:** 10.1172/JCI154619

**Published:** 2022-06-15

**Authors:** Sylvia J. Gasparini, Karen Tessmer, Miriam Reh, Stephanie Wieneke, Madalena Carido, Manuela Völkner, Oliver Borsch, Anka Swiersy, Marta Zuzic, Olivier Goureau, Thomas Kurth, Volker Busskamp, Günther Zeck, Mike O. Karl, Marius Ader

**Affiliations:** 1 Center for Regenerative Therapies Dresden (CRTD), Center for Molecular and Cellular Bioengineering, Technische Universität, Dresden, Germany.; 2 Natural and Medical Sciences Institute at the University of Tübingen, Reutlingen, Germany.; 3 German Center for Neurodegenerative Diseases (DZNE), Dresden, Germany.; 4 University of Bonn, Department of Ophthalmology, Bonn, Germany.; 5 Sorbonne Université, Institut de la Vision, INSERM, CNRS, Paris, France.; 6 Technology Platform, Center for Molecular and Cellular Bioengineering (CMCB), Technische Universität Dresden, Dresden, Germany.; 7 Institute of Biomedical Electronics, Technische Universität Wien, Vienna, Austria.

**Keywords:** Stem cells, Transplantation, Human stem cells, Retinopathy, Stem cell transplantation

## Abstract

Once human photoreceptors die, they do not regenerate, thus, photoreceptor transplantation has emerged as a potential treatment approach for blinding diseases. Improvements in transplant organization, donor cell maturation, and synaptic connectivity to the host will be critical in advancing this technology for use in clinical practice. Unlike the unstructured grafts of prior cell-suspension transplantations into end-stage degeneration models, we describe the extensive incorporation of induced pluripotent stem cell (iPSC) retinal organoid–derived human photoreceptors into mice with cone dysfunction. This incorporative phenotype was validated in both cone-only as well as pan-photoreceptor transplantations. Rather than forming a glial barrier, Müller cells extended throughout the graft, even forming a series of adherens junctions between mouse and human cells, reminiscent of an outer limiting membrane. Donor-host interaction appeared to promote polarization as well as the development of morphological features critical for light detection, namely the formation of inner and well-stacked outer segments oriented toward the retinal pigment epithelium. Putative synapse formation and graft function were evident at both structural and electrophysiological levels. Overall, these results show that human photoreceptors interacted readily with a partially degenerated retina. Moreover, incorporation into the host retina appeared to be beneficial to graft maturation, polarization, and function.

## Graphical abstract

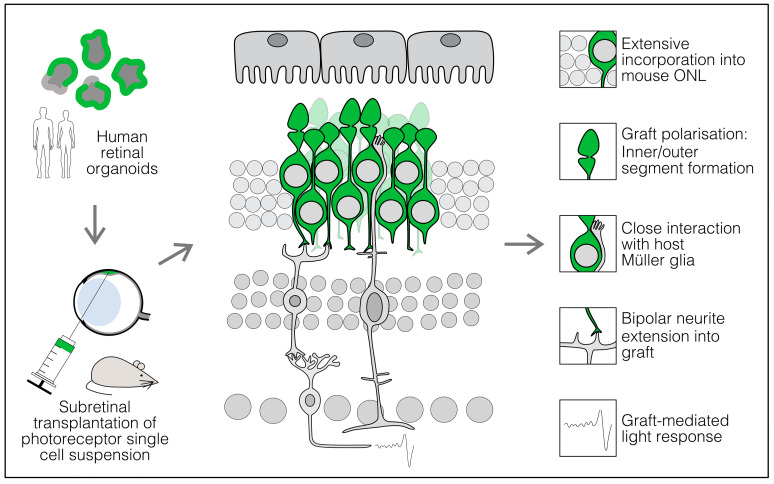

## Introduction

Vision impairment represents the most prevalent disability in the industrialized world, and very few treatment options exist (1). Many blinding diseases are characterized by the progressive loss of photoreceptor cells, which lack the ability to regenerate in mammals, including humans. Photoreceptor transplantation therapy has thus been proposed as a treatment modality in which healthy donor cells replace those that have been lost. Cell replacement therapies are an attractive option for retinal diseases, as the eye is an organ that is self-contained and partially immune privileged, minimizing the risk of unwanted cell migration and rejection (2). Additionally, the eye is readily accessible and easily monitored. The cone-rich foveal region — which is extremely important for human vision, facilitating tasks such as reading, facial recognition, and driving — is relatively small, reducing the amount of donor cells required. Within the fovea, there are only approximately 200,000 cones (3), a number of cells that is readily produced with current organoid technology. However, to date, no efficient cell-surface markers have been identified to facilitate effective sorting of donor cones. Although a marker panel for cone enrichment has been suggested, this provided low purity and yield (4).

Several recent studies have utilized human stem cell–derived retinal organoids as a source of either photoreceptors or retinal sheets for transplantation, particularly in end-stage degeneration models. While some improvements in vision have been reported through the use of retinal sheets (5–9), these grafted sheets were largely disorganized, with extensive rosette formation and the complication of donor photoreceptors mostly synapsing to donor second-order neurons rather than host cells, although synaptic connectivity has recently been improved through the reduction of bipolar cells within the organoid transplant (10). For human cone cell suspension transplantations, although functionality has recently been reported by 2 different research groups, grafts appeared disordered, with little evidence of cell polarization (11) (i.e., inner and outer segments oriented toward the retinal pigment epithelium [RPE]; axons extended toward second-order neurons), or showed some polarization but poor general transplant cell survival (12, 13).

The aforementioned studies mostly focused on transplantation into models of severe end-stage degeneration, particularly the rd1 mouse model, in which no host photoreceptors remain. However, retinal degenerative diseases are characterized by a wide variety of pathologies ranging from early to late onset, with slow to rapid photoreceptor loss (14). It is currently unknown in which retinal disease type or at what degenerative stage photoreceptor replacement therapies would be most effective. A highly degenerated environment may not be conducive to graft survival, organized graft integration, or synaptic connectivity with the host retina. In humans, extensive glial scarring and neural retinal remodeling may render end-stage transplantations challenging (15). Here, we therefore used the cone photoreceptor function loss 1 (Cpfl1) mouse, in which cones are dysfunctional and rapidly degenerate, while rods remain largely unaffected (16), in order to determine whether human cones can benefit by some means from the remaining structural support.

In this study, a cone-specific human induced pluripotent stem cell (hiPSC) GFP-reporter line was generated to facilitate FACS of an enriched cone population from retinal organoids. We used an optimized differentiation protocol that generated cone-rich retinal organoids, thus ensuring a large population of transplantable cone cells. We aimed to investigate how transplanted cones mature, as well as how the donor-host interaction changes over time after transplantation. Results showed long-term survival for up to 6 months in recipient mice, extensive and polarized incorporation into the remaining mouse outer nuclear (photoreceptor) layer, and interaction with host Müller glia and second-order neurons. Human graft incorporation was further validated through the use of donor photoreceptors from a pan-photoreceptor reporter hiPSC line. Moreover, we found that photoreceptor graft maturation and polarization were enhanced by donor-host interaction, as shown by histology, ultrastructural analysis, and transcriptomics. Human photoreceptor transplants ultimately led to the reestablishment of cone-mediated light responses in the cone-deficient mouse.

## Results

### Validation of a human cone reporter iPSC line to produce a transplantable population of human cones.

**Figure 1:**
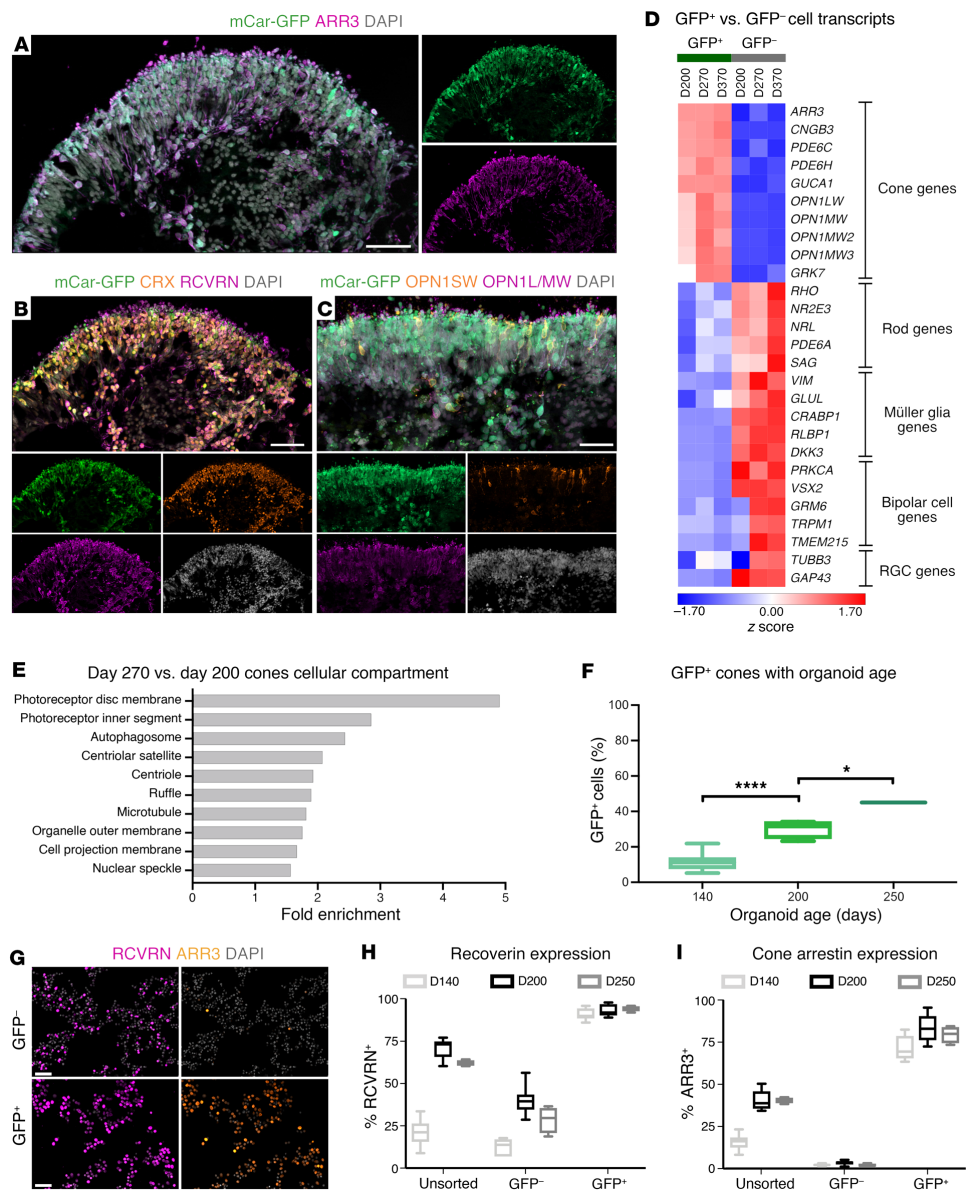
Generation and characterization of a cone-specific reporter cell line. D240 mCar-GFP–derived retinal organoid cryosections show (**A**–**C**) costaining of mCar-driven GFP with cone-specific (ARR3, OPN1SW, OPN1L/MW) and photoreceptor-specific (CRX, RCVRN) proteins. (**D**) Heatmap of *z* scores for the expression of major retinal cell–type marker genes in GFP^+^ and GFP^–^ cells sorted from mCar-GFP reporter organoids on D200, D270, and D370 after differentiation. (**E**) GO term cellular compartment overrepresentation analysis of D270 GFP^+^ cells compared with D200 GFP^+^ cells. (**F**) Proportion of GFP^+^ cells with organoid age as analyzed by immunocytochemistry. (**G**) Immunocytochemical analysis of GFP, RCVRN, and ARR3 expression in GFP^+^ and GFP^–^ FAC-sorted fractions and quantification of immunocytochemical staining of (**H**) RCVRN and (**I**) ARR3 in unsorted and GFP^+^ and GFP^–^ sorted fractions. Scale bars: 50 μm. Box-and-whisker plots indicate the upper and lower bounds from the 25th to 75th percentiles, and whiskers indicate the minimum to the maximum. Statistical significance was determined by 1-way ANOVA with Tukey’s post hoc test. *P < 0.05 and ****P < 0.0001. OPN1SW, short-wave opsin; OPN1LMW, long-/medium-wave opsin.

A hiPSC line carrying GFP under the control of the cone-specific mouse cone arrestin (mCar) promoter was generated using a piggyBac transposon system (mCar-GFP line). This did not affect the karyotype (Supplemental Figure 1, A and B; supplemental material available online with this article; https://doi.org/10.1172/JCI154619DS1). Human mCar-GFP retinal organoids were produced using a modified version of a previously published protocol that has been shown to generate robust numbers of cone photoreceptors (Supplemental Figure 1C and refs. 17–19). The mCar-driven GFP signal was predominantly located in the outer neuroepithelial layer, as would be expected for cones (Figure 1, A–C). Reporter expression colocalized with human cone arrestin 3 (ARR3) antibody staining, and all ARR3^+^ cells appeared to be GFP^+^, indicating the specificity and efficiency of this reporter (Figure 1A). The GFP^+^ cells were positive for the photoreceptor-specific markers cone-rod homeobox protein (CRX) and recoverin (RCVRN) (Figure 1B) and also expressed more mature cone markers such as long- and medium-wave opsin (L/M-opsin) and short-wave opsin (S-opsin) on day 240 (D240) of in vitro differentiation (Figure 1C). Note that we observed far more L/M-opsin cones present in the organoids than S-opsin cones, as previously described (17). Markers of other retinal cell types, namely those for rods (neural retina–specific leucine zipper protein [NRL]), Müller glia (transcription factor SOX 2 [SOX2], excitatory amino acid transporter 1 [GLAST], retinaldehyde-binding protein 1 [CRALBP]), bipolar cells (protein kinase C α type [PKCα]), and amacrine/ganglion cells (HU antigen C/-D [HUC/HUD]) did not colocalize with GFP (Supplemental Figure 1, D–F). For a more in-depth analysis of the cell identity of GFP-expressing cells, we performed next-generation sequencing (NGS) of FAC-sorted GFP^+^ and GFP^–^ cells with D200, D270, and D370 retinal organoids. This analysis confirmed that GFP^+^ cells highly expressed cone-specific genes such as ARR3, CNGB3, PDE6C, and L/M-opsins, whereas the negative fraction showed high expression of typical marker genes of other retinal cell types including rods, Müller glia, bipolar cells, and retinal ganglion cells (RGCs) (Figure 1D). Additionally, gene ontology (GO) term analysis of differentially expressed genes in cones from D200 versus D270 organoids revealed an enrichment of cellular compartment pathways critical to photoreceptor function in D270 cones, indicating that D200 cones are not yet fully mature and undergo extensive molecular changes in the following 10 weeks (Figure 1E).

To assess the proportion of cones in the organoids and the efficiency of reporter expression, we performed FACS followed by immunocytochemical analysis (Figure 1, F–I). As expected, we detected a significant increase in the proportion of GFP^+^ cells with organoid age (i.e., at D140, D200, D250), with up to 45% of cells determined to be GFP^+^ by D250 (Figure 1F). The FAC-sorted GFP^+^ fraction was found to be highly enriched in RCVRN^+^ and ARR3^+^ cells (Figure 1, G–I), whereas the GFP^–^ fraction was almost entirely depleted of ARR3^+^ cells at all time points analyzed (Figure 1I). This indicated that almost all cones were captured using the mCar-GFP reporter–based sorting system. With the confirmation of the cone identity of GFP^+^ cells, cones from D200 organoids were determined to be the most suitable population for transplantation studies, given the robust number of relatively mature cone cells present, combined with a high degree of viability following dissociation and FACS purification. We also performed a smaller transplantation study using cones from D250 organoids for comparison.

### Human cones incorporate extensively into the host retina with longer post-transplantation times.

**Figure 2:**
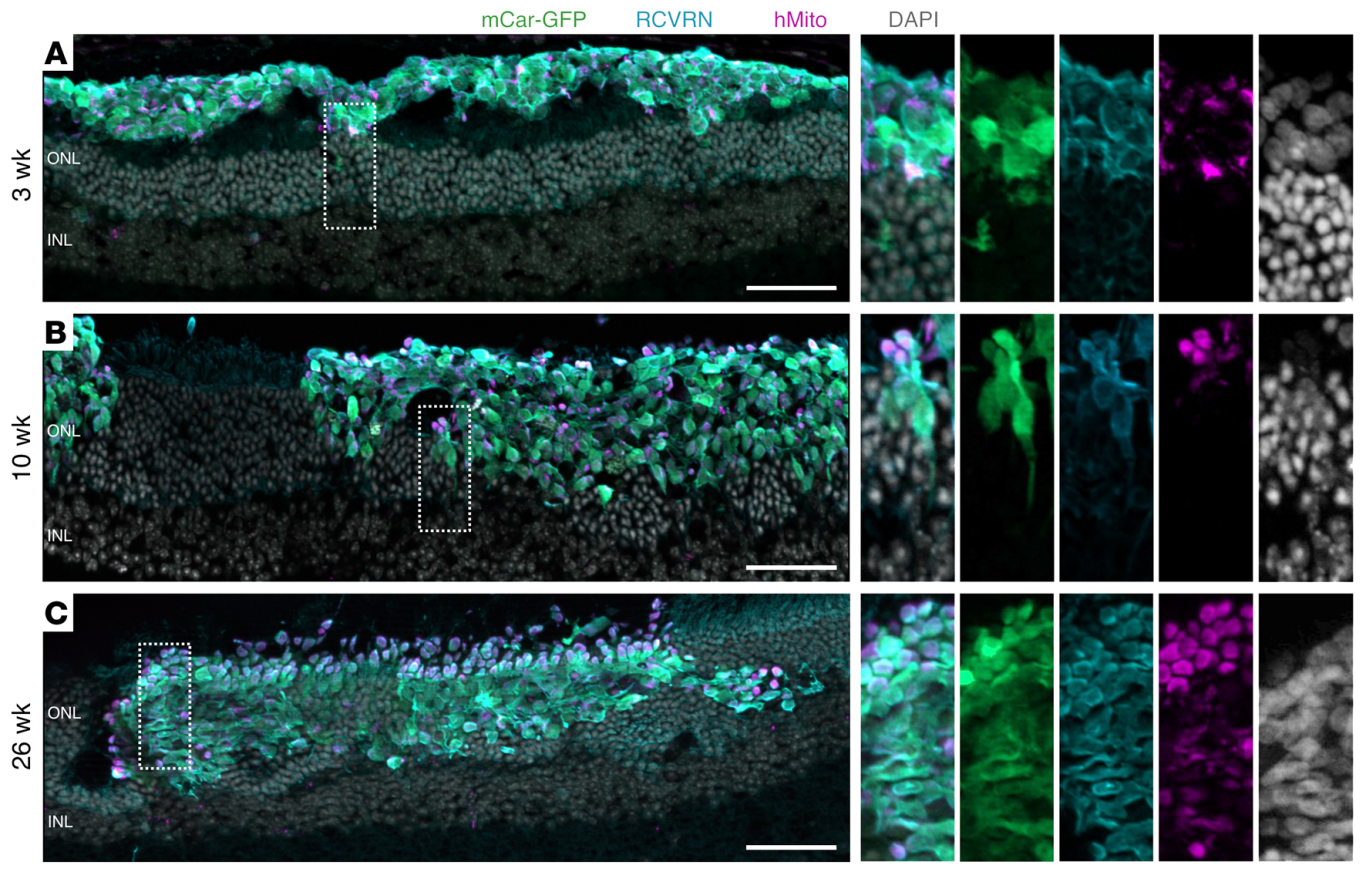
Extensive incorporation of transplanted cones into Cpfl1 host retina with increased time since transplantation. Cryosections of retina transplanted with mCar-GFP^+^ cells on post-differentiation D200 were stained for GFP, RCVRN, hMito, and DAPI and showed (**A**) minimal donor-host interaction 3 weeks after transplantation and (**B**) large cell clusters incorporated into the host retina 10 weeks after transplantation, with areas of round, mitochondria-rich outgrowths toward the RPE and axon-like extensions projected toward the INL. (**C**) By 26 weeks, the grafts displayed even more abundant mitochondria-rich outgrowths. Scale bars: 50 μm; original magnification, ×2 (insets).

Human cones were transplanted into the subretinal space (between the RPE and the photoreceptor layer) of Cpfl1 mice, which received monthly vitreal triamcinolone acetonide injections for immune suppression from the time of transplantation. All transplanted cells expressed human ARR3 across the study timeframe, and minimal immune reactivity of the host was observed (Supplemental Figure 2, A and B). Three weeks after transplantation, clusters of human cones survived in the subretinal space but did not interact extensively with the host outer nuclear layer (ONL). Donor cell clusters appeared mostly separated from the host ONL, with few contact points (Figure 2A). Strikingly, 10 weeks after transplantation, large clusters (up to 30,000 μm^2^ per retinal section) of human cones were found to be partially incorporated into the Cpfl1 host ONL (Figure 2B) and appeared to incorporate further by 26 weeks (Figure 2C). Note that this phenomenon is not due to material transfer, which is frequently observed in mouse-to-mouse photoreceptor transplantations. Here, GFP^+^ cells were identifiable as human by staining with human-specific markers for mitochondria and ARR3, as well as by their significantly larger and less dense nuclei than those of the mouse photoreceptors (Supplemental Figure 2A, see also refs. 11, 20). Additionally, transcriptome analysis by NGS confirmed the human origin of the GFP^+^ cells isolated from transplanted retinas (see below).

### Maturation of human cones within Cpfl1 hosts.

**Figure 3:**
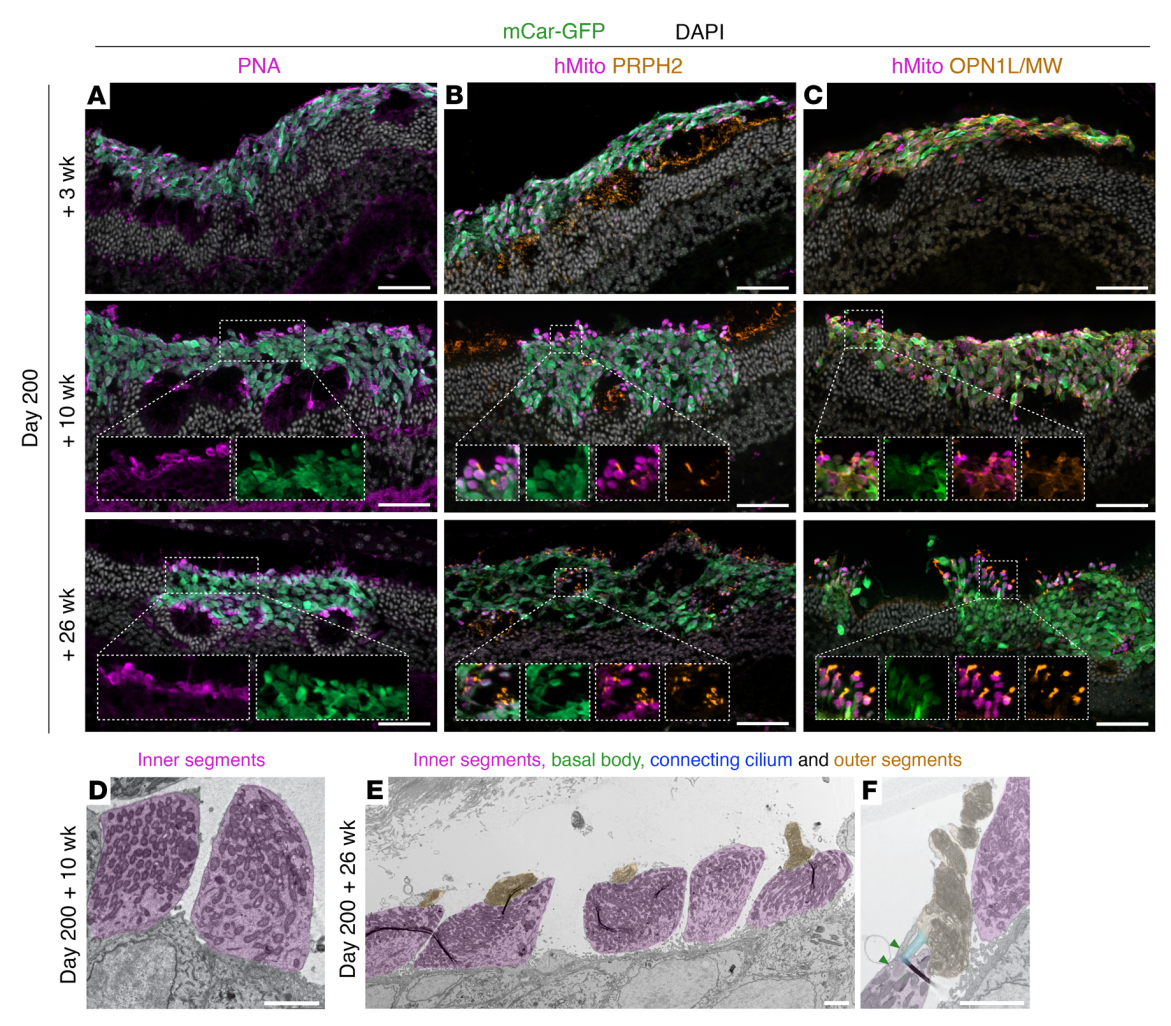
Graft development, polarization, and inner and outer segment formation. Cryosections of retina transplanted with D200 mCar-GFP^+^ cells were stained with (**A**) PNA, showing more localized PNA binding with longer post-transplantations times, (**B**) PRPH2, showing the most abundant staining 26 weeks after transplantation, and (**C**) OPN1L/MW, which also showed segment-like localization at 26 weeks. TEM of ultrathin sections of eyes transplanted with D200 cones revealed (**D**) inner segments (purple) 10 weeks after transplantation, (**E**) inner (purple) and outer (orange) segments and (**F**) occasionally basal bodies (green arrows) and connecting cilium (blue overlay) 26 weeks after transplantation. Scale bars: 50 μm (IHC images) and 2 μm (TEM images). Original magnification, ×1.5, ×2, and ×1.75 (insets in **A**, **B**, and **C**, respectively).

In addition to incorporating into the host ONL over time, human cones also appeared to further mature in vivo. Although 3 weeks after transplantation the donor cell mass was largely amorphous, by 10 weeks the transplanted cones had developed axon-like projections toward the host inner nuclear layer (INL) and mitochondria-rich bulbous outgrowths toward the RPE (Figure 2, A and B). As photoreceptor segments are characterized by 2 distinctive compartments — namely the highly metabolic inner segment containing densely packed mitochondria and the unique light-detecting outer segment, an elaborated primary cilium composed of stacked disc membranes — the observed mitochondria-rich bulbous outgrowths are indicative of inner segment development (Figure 2B). These presumed inner segments were even more widespread by 26 weeks after transplantation (Figure 2C). To confirm the inner segment identity of the bulbous mitochondria-rich outgrowths, retinal sections were stained with markers associated with inner and outer segments. Accordingly, peanut agglutinin (PNA), which is specific for cone inner segments and outer segments, was bound in a nonlocalized fashion throughout the graft at 3 weeks. By 10 weeks, and even more prominently by 26 weeks, however, the PNA label was increasingly concentrated in mitochondria-rich regions, i.e., the RPE-facing edge of incorporated grafts and the rosette-like structures, which occurred in some areas in which mouse photoreceptors remained underneath the incorporating graft (Figure 3A). Peripherin 2 (PRPH2) staining of outer segments was not evident in the human cones at 3 weeks and only occasionally at 10 weeks after transplantation (Figure 3B), while L/M-opsin was largely distributed throughout the cell body at these time points (Figure 3C). However, by 26 weeks, the expression of PRPH2 and L/M-opsin was restricted to segment-like structures, in close association with the putative inner segments (human mitochondria [hMito]), suggestive of outer-segment formation by this time point (Figure 3, B and C).

To investigate the extent of photoreceptor maturation further, we examined grafts at the ultrastructural level. Indeed, we found many examples of inner segments 10 weeks after transplantation (Figure 3D), whereas outer segments were not found. By 26 weeks, however, numerous cones formed relatively well-organized and tightly-stacked outer segment–like structures that were sometimes found to be joined to inner segments via a connecting cilium, additionally identifiable by the characteristic basal bodies (Figure 3, E and F). The cells displaying these photoreceptor-specific features were confirmed to be of human origin by Immunogold labeling of GFP and human-specific ARR3 (Supplemental Figure 3, A and B), as well as through the distinctive size and morphology of the human cone nuclei, which are much larger and less electron dense than the mouse photoreceptor nuclei (magnified insert in Figure 2A and Supplemental Figure 3C).

**Figure 4:**
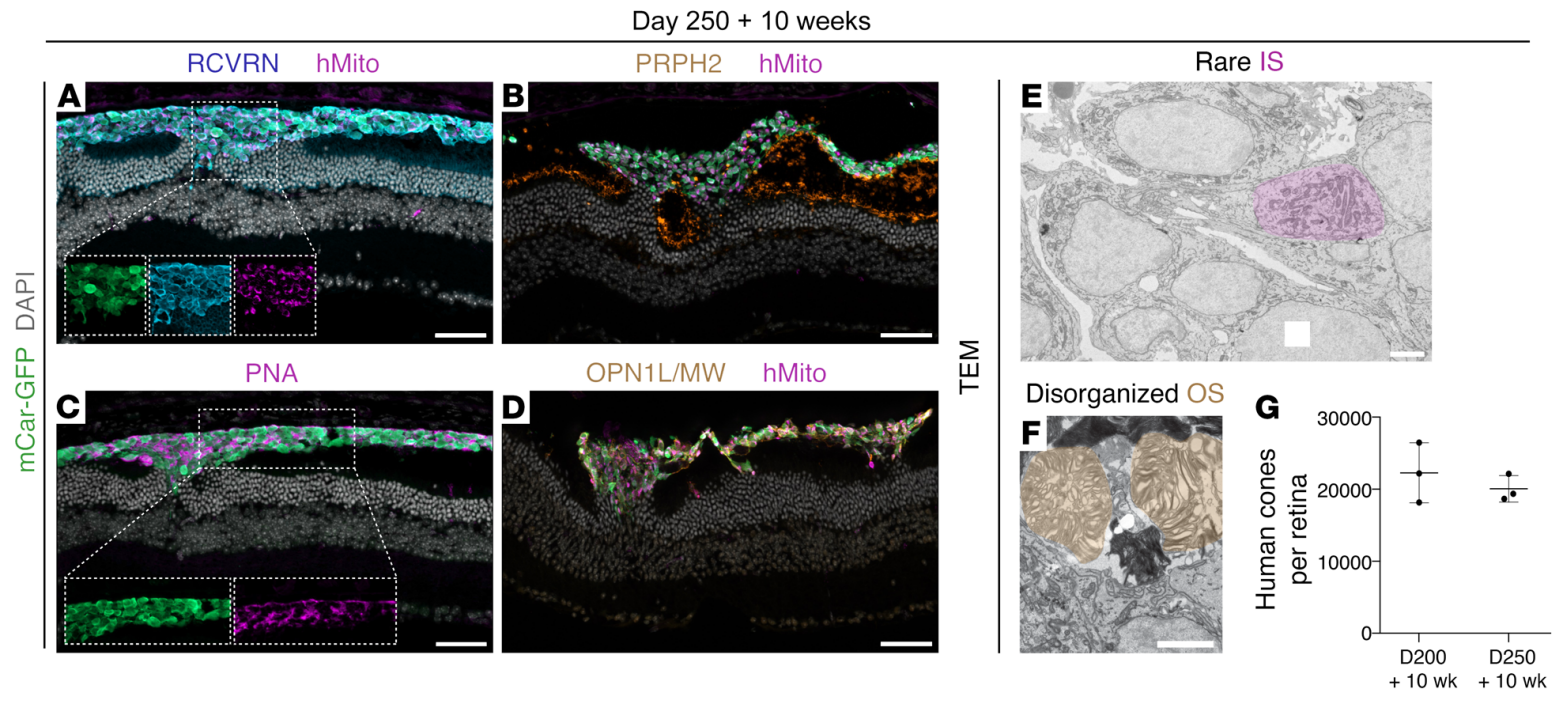
Minimal donor-host interaction, polarization, or maturation in D250 grafts. Cryosections of retina transplanted with mCar-GFP^+^ cells on D250 after differentiation showed (**A**) minimal donor-host interaction and few mitochondria-rich outgrowths, (**B**) little PRPH2 staining, (**C**) dispersed PNA binding, and (**D**) mislocalized L/M-opsin staining. TEM of the D250 transplanted cones showed (**E**) few inner segments (IS) and (**F**) occasional disorganized outer segments (OS). (**G**) A similar number of cells (~20,000 cells) survived in D200 plus 10-week and D250 plus 10-week samples. Scale bars: 50 μm (IHC images) and 2 μm (TEM images).

As inner segments and particularly outer segments took a long time to develop after transplantation, we postulated that transplanting cones derived from older organoids might reduce the time required for the in vivo development of such mature photoreceptor-specific features. Cones isolated from D250 retinal organoids were transplanted and assessed 10 weeks after transplantation. Interestingly, unlike D200 cones, after 10 weeks in vivo, most of the D250 grafts remained in the subretinal space, indicating a reduced capacity of the older cells to incorporate into the host ONL (Figure 4A). Much like D200 plus 3-week transplantations, the D250 plus 10-week grafts presented as a largely amorphous cell mass with few mitochondria-rich, L/M-opsin, or PRPH2 outgrowths, and PNA or L/M-opsin labels dispersed throughout the cell mass, rather than accumulating toward the RPE (Figure 4, B–D). At an ultrastructural level, we observed occasional inner segments as well as some outer segments, however, the outer segments were highly disorganized and not tightly stacked (Figure 4, E and F). Although photoreceptors of D250 plus 10-week grafts (i.e., post-differentiation D320) were in total older than D200 plus 10-week grafts (post-differentiation D270), they, in comparison, show a decreased capacity for incorporation and maturation. Of note, there was a similar degree of cell numbers after transplantation (~20,000 cells per eye) with D200 and D250 transplantations, thus, the differences in incorporation did not seem to arise from differential cell survival with donor organoid age (Figure 4G). This suggests that D200 cones were a preferable donor cell age, and that, together, these observations indicate that donor cone age and time in vivo are important factors for transplant incorporation and maturation.

### Incorporating cones show close interaction with host Müller glia.

In normal retinal physiology, photoreceptors are intermingled in a dense network of Müller glia processes that support photoreceptor structure, homeostasis, and function. Müller glia, for example, participate in the cone visual cycle (21), and, together with photoreceptor inner segments, seal the neuroretina from the subretinal space through the outer limiting membrane (OLM), a continuous band of heterotypic adherens junctions. Therefore, we assessed the interaction between transplanted human cones and recipient Müller glia.

**Figure 5:**
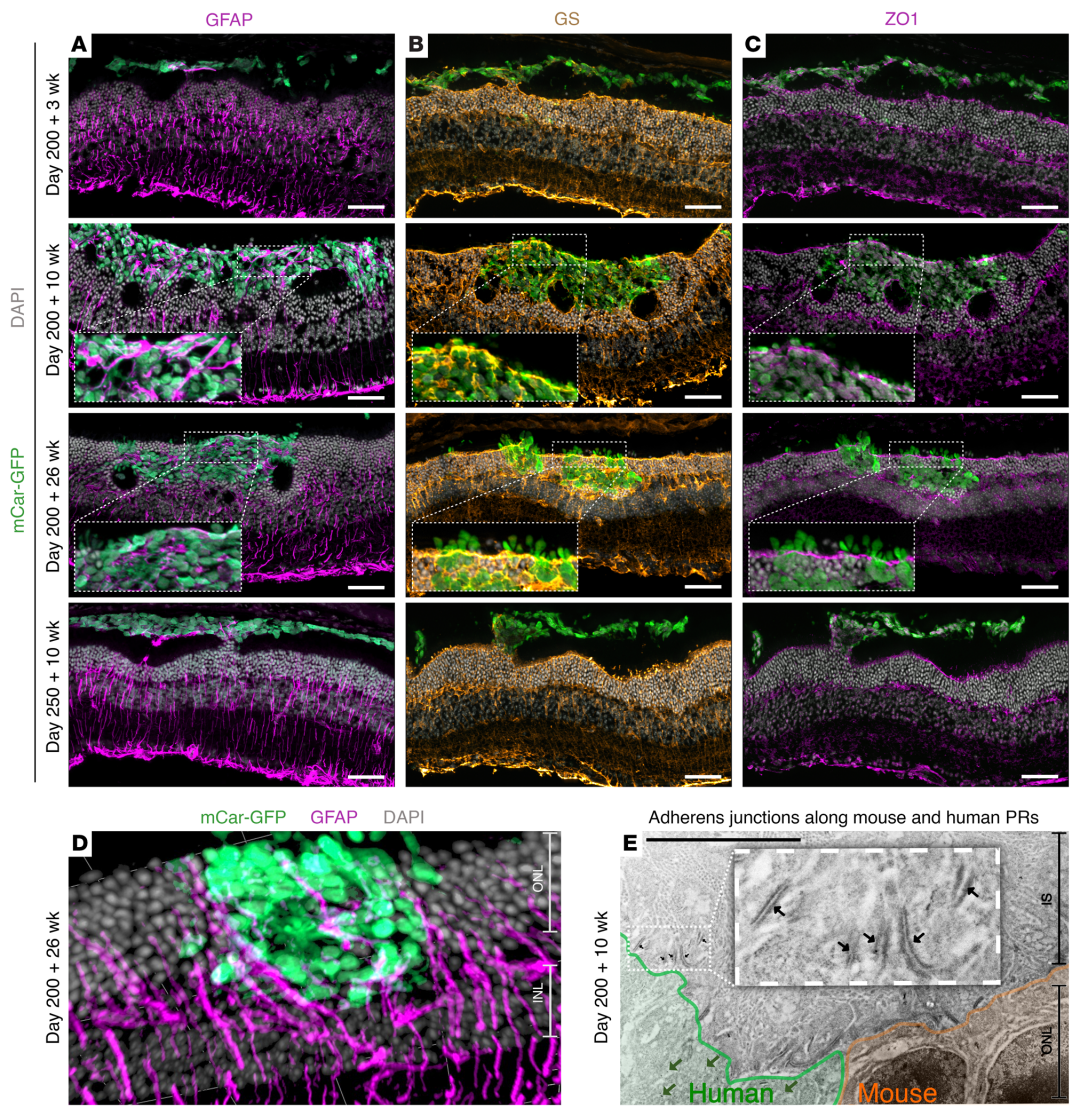
Host Müller glia interaction with human cone grafts. Cryosections of retina transplanted with mCar-GFP^+^ cells on D200 or D250 after differentiation showed (**A**) Müller glia beginning to extend processes into areas where the graft contacted the host ONL (D200 plus 3 weeks, D250 plus 10 weeks) and extensive intermingling with grafts (D200 plus 10 and D200 plus 26 weeks), which had incorporated into the host ONL. (**B**) GS and (**C**) ZO1 staining indicated that an outer limiting membrane–like structure formed between the subretinal space and donor cell nuclei incorporating the human cones into the host ONL. (**D**) 3D reconstruction of GFAP^+^ Müller glia processes extending around human cones. (**E**) Immunogold labeling confirmed the formation of a series of adherens junctions between mouse and human photoreceptors. Dark green arrows indicate examples of Immunogold 10 nm labeling of human ARR3, and black arrows indicate adherens junctions between mouse Müller glia processes and both mouse and human photoreceptors. Scale bars: 50 μm (IHC images), 6 μm (CLEM images), and 50 μm (3D reconstruction grid lines). Original magnification, ×2.25 (**A**–**C**) and ×3.25 (**E**). PR, photoreceptors.

Immunohistochemical staining for glial fibrillary acidic protein (GFAP) revealed that, in the D200 plus 3-week and D250 plus 10-week transplants, Müller glia processes extended into the graft only in limited areas where donor clusters started to make contact with the ONL, whereas no GFAP staining was observed within subretinal-located graft areas (Figure 5A). By D200 plus 10 weeks, rather than forming a glial barrier, we found that Müller glia processes permeated throughout the graft (Figure 5A). Further staining with glutamine synthetase (GS), zonula occludens protein 1 (ZO1), and phalloidin indicated the formation of an OLM-like structure in between the human nuclei and the subretinal space (Figure 5, B and C, and Supplemental Figure 4A). The actin-dense, ZO1^+^ and GS^+^ band above the human nuclei is continuous and in line with the host OLM and seemingly incorporates the clusters of human cones rather than excluding the xenogeneic cells. This interaction was maintained at 26 weeks (Figure 5, A–D, and Supplemental Figure 4A).

These observations were confirmed by electron microscopy (EM), in which close association of Müller glia processes and human cones was evident. The continuous band of adherens junctions formed between the human cones and mouse rods at the base of their inner segments is reminiscent of an OLM structure (Figure 5E).

**Figure 6:**
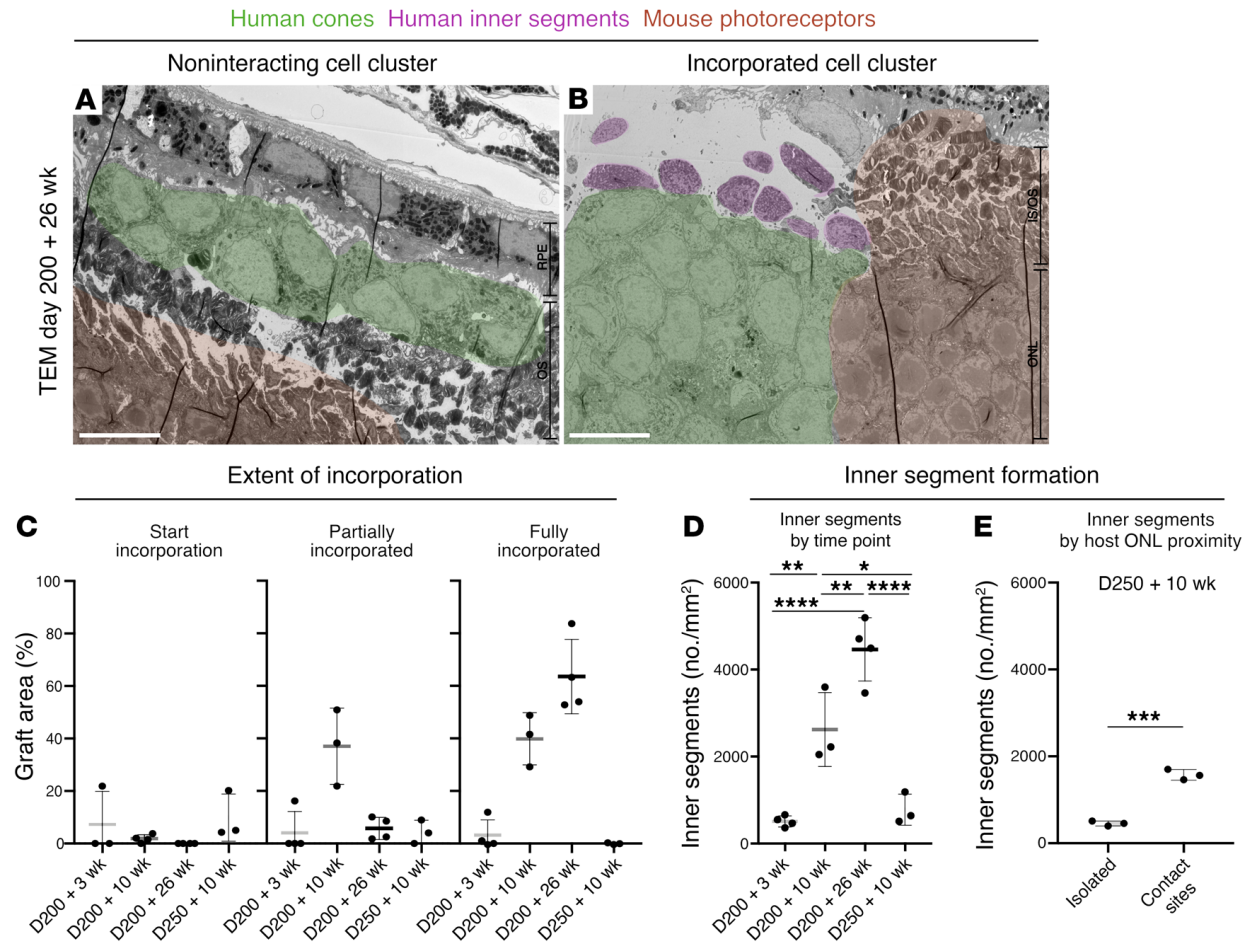
Interactive grafts more readily develop inner segments. Representative TEM images of ultrathin retinal sections, in which some cone clusters (green overlay) within the same mouse eye (**A**) remained in the subretinal space or (**B**) incorporated into the host ONL (mouse photoreceptors, orange overlay) and developed inner segments (purple overlay). (**C**) Quantification of retinal cluster interaction with the host retina by area (*n =* 3–4 eyes). (**D**) Number of mitochondria-rich presumed inner segments at each time point (*n =* 3–4 eyes). (**E**) Increased inner segment formation in interactive versus isolated areas in D250 plus 10-week grafts. Scale bars: 10 μm. Data are displayed as the mean ± SD. *P < 0.05, **P < 0.01, ***P < 0.001, and ****P < 0.0001. Statistical significance was determined by 1-way ANOVA with Tukey’s post hoc test.

Importantly, we also observed that, even within the same eye, it was primarily in clusters of incorporated human cones that mature photoreceptor-specific features of inner and outer segments developed (Figure 6B), whereas those clusters of cones that remained isolated in the subretinal space without obvious interaction with host Müller glial processes persisted largely amorphously (Figure 6A).

To quantify the extent of donor-host interaction at different experimental time points, the total transplanted cell area was determined, and the percentage of interacting grafts was calculated. Here, the apical border of the host ONL was used to define incorporation. Grafts with 5%–20% of the transplant within the apical border were classified as starting to incorporate, 20%–80% as partially incorporated, and only those with 80%–100% were considered fully incorporated (Supplemental Figure 5). Approximately 40% of the D200 plus 10-week transplant cell cluster area partially incorporated and a further 40% fully incorporated into the host ONL (Figure 6C). By D200 plus 26 weeks, over 60% of the graft area was fully incorporated. Both the D200 plus 3-week and the D250 plus 10-week samples only minimally interacted with the host retina (~85% graft area noninteracting) (Figure 6C). Accordingly, only D200 plus 10-week and D200 plus 26-weeks grafts exhibited numerous mitochondria-rich outgrowths, i.e., inner segments (Figure 6D). If this were simply a factor of cell age, one would expect D250 plus 10-week grafts to display at least as many inner segments as D200 plus 10-week grafts, however, in line with our previous observations, these developed very few mitochondria-rich inner segments. Moreover, where inner segments did develop, we observed that these appeared almost exclusively in areas where the host retina was directly contacted by the graft rather than in isolated grafts. Upon quantification, it was established that 3 times as many inner segments developed in regions of the D250 plus 10-week grafts contacting the host ONL versus isolated graft areas in the same eye (Figure 6E), again indicating that interaction with the host influenced the maturation and development of photoreceptor-specific morphological features like inner segments.

### Cones mature more extensively in the mouse retinal environment compared with those maintained in retinal organoids in vitro.

**Figure 7:**
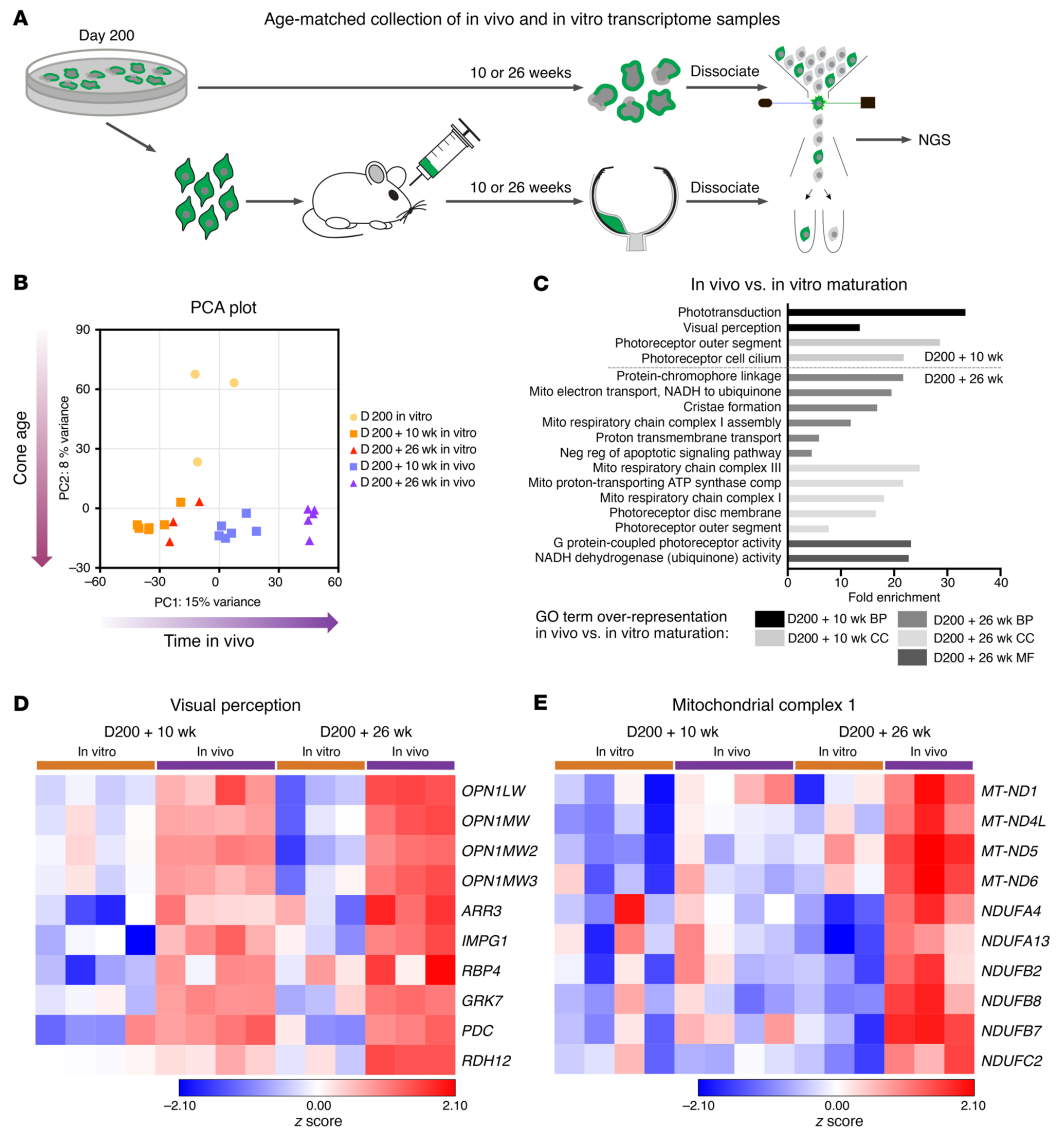
Transcriptional profiling of transplanted cones compared with age-matched, organoid-derived cones. (**A**) Schematic representation of the mCar-GFP^+^ cone sequencing workflow. (**B**) PCA of the top 500 differentially regulated genes. (**C**) GO term pathway overrepresentation analysis of in vivo–matured versus in vitro–matured cones. BP, biological process; CC, cell compartment; MF, metabolic function. Heatmaps of *z* scores for genes involved in (**D**) visual perception and (**E**) mitochondrial complex 1.

In order to further investigate whether the maturation trajectory of the retinal organoid–derived cones was influenced, as we suggest, by the host retinal environment, we compared the transcriptional profile of transplanted cones with cones from age-matched retinal organoids. D200 organoids were maintained for a further 10 or 26 weeks (henceforth referred to as in vitro), and transplanted whole eye cups were collected 10 and 26 weeks after transplantation (hereafter referred to as in vivo). Both in vitro and in vivo samples were dissociated, and GFP^+^ cells were collected via FACS for RNA-Seq (Figure 7A). Interestingly, PCA analysis of the top 500 differentially regulated genes revealed that the greatest source of variance in the data separated clusters not according to their age (D200 plus 10-week and D200 plus 26-week in vitro samples clustered closely together in PC1), but according to the time in vivo, indicating that maturation within the host retina indeed played an important role (Figure 7B). More detailed gene overrepresentation analysis showed that molecular mechanisms, biological processes, and cellular compartment pathways involved in light perception were highly and significantly enriched in the in vivo–matured cone samples (Figure 7C). Both L/M-opsins as well as other outer segment–related genes were highly upregulated in the in vivo–matured samples — particularly after 26 weeks (Figure 7D). To complement this analysis, we performed EM and IHC analyses in age-matched organoids. No localization of L/M-opsin to segment-like structures was evident in the D370 organoids (Supplemental Figure 6), unlike what we observed in the D200 plus 26-week transplants (Figure 3C). Accordingly, EM analysis revealed that photoreceptors in organoids did not develop the nicely stacked discs evident in D200 plus 26-week transplants (Supplemental Figure 6). In the D200 plus 26-week in vivo cones, we also observed enrichment in many mitochondrial and respiratory pathways compared with age-matched, in vitro–matured cones, indicating a higher metabolic capacity in the in vivo–matured cones (Figure 7, C and E). This analysis supports the histological and ultrastructural evidence that the host’s retinal environment promoted the maturation of organoid-derived human cones, leading to enhanced inner and outer segment formation, which is critical to light detection.

### Validation of donor cell incorporation using the Crx-mCherry iPSC cell line.

**Figure 8:**
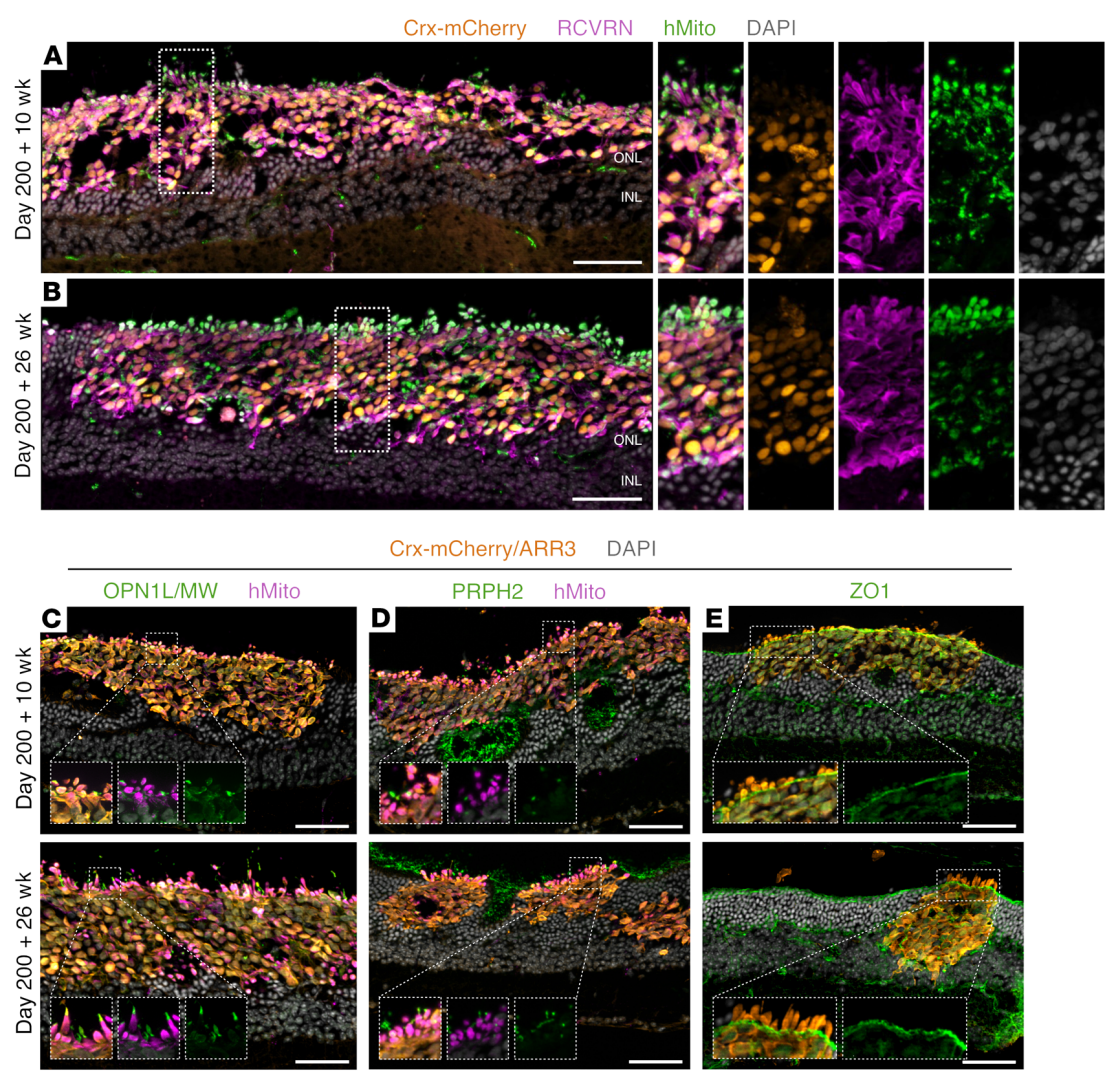
Crx-mCherry^+^ grafts also display extensive incorporation and polarization. Retinal cryosections of Crx-mCherry^+^ grafts were transplanted on D200 and stained with RCVRN, hMito, or DAPI. (**A**) By 10 weeks, large cell clusters incorporated into the host retina with areas of round, mitochondria-rich outgrowths toward the RPE and axon-like extensions projected toward the INL (see magnified insets in **A**). (**B**) By 26 weeks, the grafts displayed even more abundant mitochondria-rich outgrowths. (**C**) OPN1L/MW and (**D**) PRPH2 were more extensively expressed in segment-like structures 26 weeks after transplantation. (**E**) Müller glia processes formed a ZO1^+^ outer limiting membrane–like structure incorporating transplanted cells into the host ONL. Scale bars: 50 μm. Original magnification (insets), ×1.5 (**A** and **B**), ×2 (**C**–**E**).

To determine whether the incorporating capacity displayed by the human cones was specific to this cell line, we generated and transplanted photoreceptors from a Crx-driven mCherry reporter iPSC line (22). CRX is expressed in retinal progenitors, rods, and cones, with Crx-mCherry thus marking both photoreceptor cell types (Supplemental Figure 7). FAC-sorted D200 Crx-mCherry^+^ cells were transplanted into Cpfl1 mice as per the mCar-GFP^+^ cones. We observed a remarkably similar phenotype, in which Crx-mCherry^+^ photoreceptor transplants appeared to replace whole sections of mouse ONLs (Figure 8, A and B), with apical-oriented inner and nascent outer segments (Figure 8, A–D). Also, the formation of a ZO1^+^, OLM-like band between nuclei and inner segments was again evident, with Müller glia pervading and seemingly incorporating the graft (Figure 8E and Supplemental Figure 4, B and C).

### Evidence for contact between host second-order neurons and transplanted human cones.

**Figure 9:**
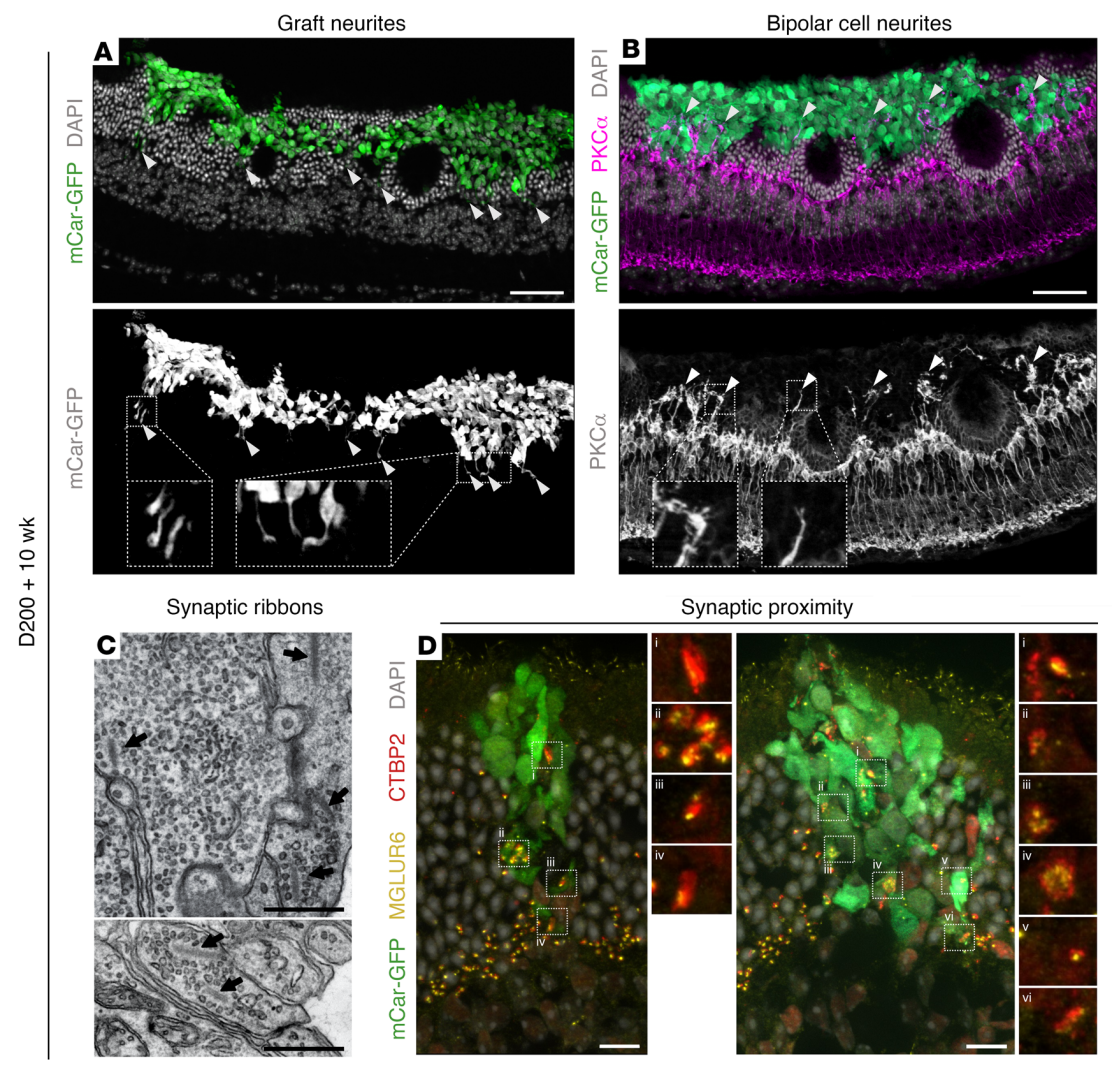
Putative synapse formation between transplanted human cones and host bipolar cells. Immunolabeled cryosections of Cpfl1 retina transplanted with mCar-GFP^+^ cells show (**A**) neurite extension from grafted cells toward the host INL and (**B**) widespread neurite extension into the cone cell graft from PKC^+^ host rod bipolar cells. White arrowheads indicate areas of neurite extension. (**C**) Representative ribbons and vesicles, components of the photoreceptor presynapse, highlighted by arrowheads in a TEM image of an incorporated graft. (**D**) Close association of the presynaptic ribbon synapse marker CTBP2 and the bipolar postsynaptic marker MGLUR6. Scale bars: 50 μm (IHC images) and 500 nm (TEM images). Original magnification (insets), ×3 (**A** and **B**), ×2.75 (**D**).

Next, we aimed to assess whether there is also synaptic connectivity between transplanted photoreceptors and host second-order neurons in the highly interactive grafts. Indeed, we observed cone axon–like protrusions projecting from the graft toward the host INL (Figure 9A), and the presence of typical human photoreceptor presynaptic ribbons was already confirmed by EM 10 weeks after transplantation (Figure 9C). On the postsynaptic side, immunohistochemical staining showed that both PKCα^+^ rod bipolar cells and secretagogin^+^ cone bipolar cell neurites extended extensively into human cone clusters in areas where the donor cells were incorporated into the host ONL (Figure 9B and Supplemental Figure 8A). Similarly, horizontal cells stained by calbindin also extended neural processes into the human cone grafts (Supplemental Figure 8B). To further investigate connectivity between donor and host cells, an association between pre- and postsynaptic markers was assessed. As seen in Figure 9D, many examples of ribbon synapses labeled by C-terminal–binding protein 2 (CTBP2) within the graft could be found in close proximity to the bipolar cell postsynaptic marker metabotropic glutamate receptor 6 (MGLUR6). These observations indicate a putative synaptic connectivity between graft and host.

**Figure 10:**
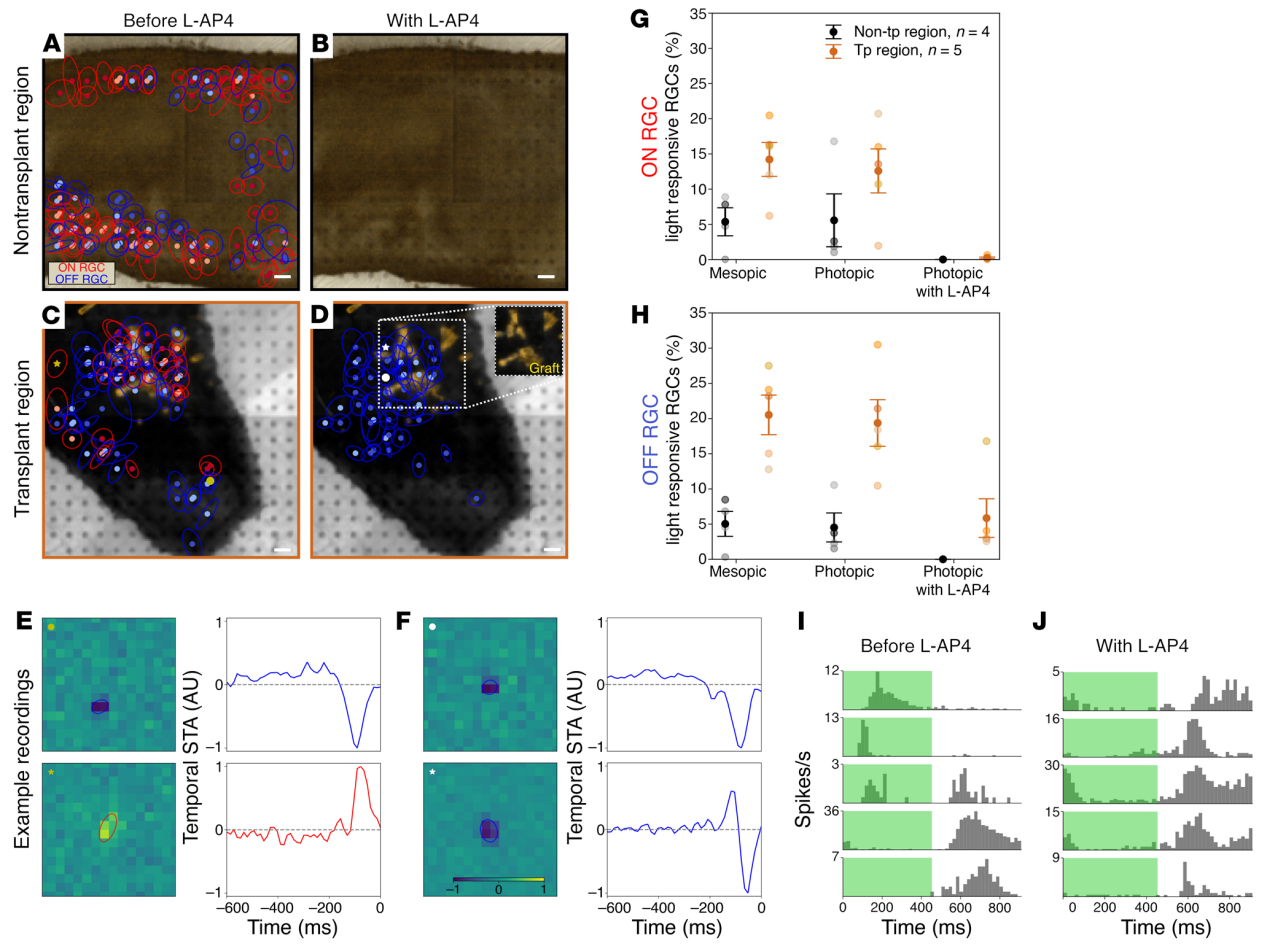
Increased RGC activity after Crx-mCherry^+^ photoreceptor transplantation. Receptive fields for ON and OFF RGCs detected following photopic stimulation in (**A**) nontransplant-containing control regions and (**C**) retinal areas containing Crx-mCherry grafts. (**B**) No photopic response was detected after addition of L-AP4 in nontransplant-containing retina. (**D**) OFF RGC cone pathway responses remained following photopic stimulation and addition of L-AP4 in retinal areas containing Crx-mCherry grafts. (**E**) Example receptive fields and temporal spike-triggered average (STA) for the 2 cells labeled with a yellow asterisk and a yellow circle in **C**, showing both ON and OFF responses. (**F**) Example receptive fields and temporal STA for the 2 cells labeled with white asterisk and a white circle in **D**, where only OFF responses remained. Percentage of light-responsive (**G**) ON RGCs and (**H**) OFF RGCs detected under mesopic, photopic, and photopic stimulation with the addition of L-AP4. Response of 5 different RGCs during full-field photopic ON-OFF flicker stimulation (**I**) before (ON, OFF, and ON-OFF RGC responses) and (**J**) with L-AP4 treatment (OFF RGC responses only). The bin width is 20 ms, and a total of 120 stimulus repetitions were performed. Scale bars: 200 μm. Original magnification, ×0.8 (inset in **D**).

To evaluate the functionality of these potential connections, we performed electrophysiological measurements using multielectrode array (MEA) recordings. Here, because of technical challenges associated with cell mass localization of GFP causing severe bleaching, we used retinas containing Crx-mCherry cells. Robust and stable ON and OFF photopic light–evoked responses (30 minutes of binary checkerboard white noise stimulation with stringent spike threshold settings to reduce artifacts) were detected in 5 of the 9 transplanted eyes tested (Figure 10C). However, low levels of photopic light responsiveness were also detected in nontransplanted regions of the same retina (Figure 10A), but only following fluorescence stimulation (Supplemental Figure 9B), which was necessarily applied to locate the cell mass. Rods have been reported to respond to photopic light when oversaturated (23). To eliminate potential endogenous oversaturated rod activity, we added the metabotropic glutamate receptor blocker L-2-amino-4-phosphonobutyric acid (L-AP4) during recording (Figure 10, B and D). L-AP4 blocks synaptic transmission between photoreceptors and all ON bipolar cells, including rod bipolar cells. Spike-triggered averaging was then used to categorize the ganglion cell response types (Figure 10, E and F). As expected, L-AP4 effectively quenched all ON RGC responses (Figure 10, G–J). Moreover, OFF responses, which are driven by cone bipolar cells, remained only in the samples containing transplanted cells (Figure 10, D and H), which is strong evidence that the light-induced spiking activity was driven by the transplanted photoreceptors because of the lack of functional endogenous cones. Retinal tissue collected and stained after recording did not show any evidence of material transfer, thus cell support was not likely the cause of functional improvement (Supplemental Figure 9). Note that when the receptive field of the active ganglion cells was calculated, there was a high degree of overlap with the cell mass location (Figure 10D), further indicating that the transplant was driving the functional response to photopic light.

## Discussion

In this study, a human cone–specific GFP reporter iPSC line facilitated the efficient enrichment of human cone photoreceptors from retinal organoids. The use of a local immune suppressant, monthly vitreal injection of triamcinolone acetonide, prevented the rejection of these human cells when transplanted into the Cpfl1 mouse subretinal space. This allowed long-term follow up over a 6-month period (26 weeks). With longer transplantation times, grafts interacted extensively with the host retina. These findings were confirmed through transplantations of a second photoreceptor-specific reporter hiPSC line. Rather than forming a glial barrier, Müller glia intermingled throughout the graft, leading to the establishment of a series of adherens junctions between mouse and human cells. Second-order neurons extended neurites into the transplant, forming potential synaptic connections. The incorporation of transplanted human cones into the host retina was accompanied by an improvement in cell polarization and maturation of photoreceptor-specific morphological features, namely inner and outer segments. The light-detecting capacity and putative synaptic connectivity of transplanted human photoreceptors were further supported by light-evoked electrophysiological recordings of downstream RGCs.

Human photoreceptor– and rod-specific embryonic stem cell/iPSC (ESC/iPSC) reporter lines have been previously generated (22, 24–27), and a very recent study produced a cone-specific human ESC line (28), however, to our knowledge, no cone reporter hiPSC line has been reported. Based on immunohistochemical and transcriptional assessment, the mCar-GFP iPSC reporter line presented here appeared to robustly and specifically label human cone photoreceptors. This is not only useful for transplantation studies, but may also be of interest in other applications, e.g., studying human cone development or in the identification of human cone–specific cell-surface markers. A previous study used viral labeling of L/M-opsin cones to allow identification of cone cell-surface markers. Not only does this exclude S-cones, but also, because of the viral transduction efficiency, only about half of the total cone population was labeled (4). The resulting marker panel led to a maximal enrichment of approximately 50% of the cones. A pan-cone reporter line would be of use in this context, as the identification of cell-surface markers is highly advantageous in a clinical setting, where reporter or virus-labeled fluorescent cells cannot be used.

In this study, we show extensive incorporation of human cones and Crx-mCherry^+^ photoreceptors into the mouse ONL. To our knowledge, this is the first report of such extensive incorporation of donor photoreceptors into the host retina from any species. Mouse-into-mouse photoreceptor transplantations largely result in material transfer rather than structural integration (29–33) — a mechanism that was ruled out in this study. This is not to assume that in a human-to-human transplant, material transfer can be discounted. In early studies utilizing younger human photoreceptors, the apparent integration and polarization of individual donor cells was not controlled for potential material transfer or viral reporter mislabeling (34, 35). It is feasible that the xenogeneic context or donor population used in the present study were not conducive to material transfer, and material transfer may yet prove to be a potential option for cell support therapy under different conditions.

The incorporative phenotype we observed was striking and distinct from results in other publications. Yet, as most recent studies of human photoreceptor suspension transplantation were either performed over a shorter time period and/or focused on transplantation into a fully degenerated retina (11–13, 20, 22), those experiments would not be expected to result in the aforementioned incorporation because of the insufficient amount of time (at 3 weeks, only limited interaction was seen) or the lack of ONL in which to incorporate. While 3 weeks after transplantation donor grafts mainly remained in the host’s subretinal space with only few contact points to the host ONL, extensive incorporation was evident starting 10 weeks after transplantation onward. With longer post-transplantation times, and particularly with smaller clusters, human cones often fully incorporated into the host ONL, seeming to replace stretches of host photoreceptors with no obvious physical impediment to the host INL.

However, areas where some host photoreceptors remained below the graft formed rosette-like structures reminiscent of outer retinal tubulations. Such tubulations are a well-known pathology upon retinal degeneration or damage (36), and it is assumed that this arrangement has a beneficial effect on photoreceptor survival when the RPE is defective. In the present case, rosette formation might therefore have been a response to the inaccessibility of RPE support in instances when the graft was located in between the ONL and the RPE. Host degeneration, such as rosetting, may even be beneficial for donor cell incorporation. Intriguingly, Müller glia outgrowth into the subretinal space upon retinal detachment is closely correlated with cone interactions, thus, upon damage, the transplanted cones may be secreting a factor to promote Müller glia remodeling and in turn incorporation (37).

Single-cell suspension studies have often been criticized for the lack of structure of the resulting graft (38). Although, in theory, retinal sheet transplantation could provide preestablished structure, current studies have described extensive rosette formation in the graft and self-synapsing to graft second-order neurons (5, 7–9, 39, 40), although a recent study provides evidence of improved synapse formation when organoid bipolar cell numbers are reduced (10). Sheet transplantations are surgically more challenging, particularly in the context of degenerative retinas, in which rupture of the tissue remains a potential risk. In this study, however, prepurification of the transplanted cells was possible because of our photoreceptor-specific reporter lines and the suspension technique we used. Unlike in other studies, the incorporated cones and Crx-mCherry^+^ photoreceptors appeared to become well polarized, with axon-like projections toward the INL and the inner and outer segments toward the RPE. As photoreceptor loss is not complete until very late stages of blinding diseases, the remaining ONL may, as in this study, provide a structural framework for more organized integration of transplanted photoreceptors. This structural organization is likely aided by the close interaction with the host Müller glia cells.

In the present work, graft maturation capacity was only observed upon incorporation into the host ONL. Through recovery of transplanted cells for NGS — a technique that has not yet been applied to photoreceptor transplants — we could show that in vivo–matured cones from time points with extensive incorporation had significant upregulation of visual transduction and outer segment–related genes when compared with age-matched organoids in vitro. This was supported histologically by the lack of equivalent outer segment formation in age-matched organoids. With longer post-transplantation times, in vivo–matured cones also increasingly expressed mitochondria-associated genes, which is noteworthy, as mature cones are known to have very high energy requirements (41). Graft incorporation and maturation were further accompanied by close interaction with Müller glia, which not only intermingled throughout inner segment developing clusters, but even participated in forming a series of adherens junctions between human and mouse photoreceptor areas. Whether the Müller glia directly enhance maturation of the transplant remains to be proven, however, it is well known that glia are important supporters of neuronal function. Müller glia are critical for photoreceptor neurite outgrowth in both 2D and 3D culture systems (42, 43). Interestingly, Müller cells are also reported to play a role in organized outer segment assembly (44). In the present study, we observed highly disorganized outer segments that developed within older (D250) cone grafts, which incorporated to a much lesser extent and did not show much interaction with host Müller glia processes.

While several studies have shown evidence of nascent outer segment formation — often in the organoids before transplantation rather than in the graft itself — these are usually small and/or have limited, disorganized discs (20, 45–49). A recent exception to this are the small but organized outer segments described by Ribeiro and colleagues, however, no connecting cilium was shown (11). The outer segments seen in our study (D200 plus 26 weeks) were not only tightly stacked and relatively well organized but were also sometimes seen to project from the inner segment via a connecting cilium, a feature that, to our knowledge, has not previously been reported in human photoreceptor suspension transplantations. Of note, organized outer segment formation including connecting cilium has been described in retinal sheet transplants (40), however, these formed primarily within rosettes, which would likely negatively affect function. A recent study involved transplantation of optogenetically engineered photoreceptors to circumvent the necessity for outer segment formation (50). Although restored visual function was observed, this was limited to the specific wavelength of the optogene and had kinetics different from that of normal visual perception. A better understanding and ideally modification of the factors required to encourage transplanted photoreceptors to develop and correctly form distinctive outer segment structures critical for light detection is of great importance if photoreceptor cell replacement therapy is to be an effective treatment modality.

Further interaction of host and donor tissue was seen at the level of the second-order neurons. Rod and cone bipolar cells as well as horizontal cells extended neurites into the transplant. The close proximity of pre- and postsynaptic ribbon synapse proteins supports the putative formation of synaptic connections. A similar plasticity in second-order neurons was already described in rd1 mice upon photoreceptor transplantation (11), but it is interesting that this effect is also seen in the Cpfl1 host, where rod photoreceptors largely remain. This implies that the incorporated cell mass can replace existing connections, as we observed dendritic remodeling of host second-order neurons only in areas of human cone incorporation. Of note, the apparent synaptic connectivity occurred already at 10 weeks, preceding the extensive maturation of donor cells, as is also seen during development. Whether signaling from second-order neurons is involved in the improved maturation of donor photoreceptors upon host interaction is an interesting avenue to pursue in future studies.

In the aforementioned study, photopic light–evoked responses by MEA were also reported (11). In our context, MEA recordings were complicated by the oversaturation of endogenous rods due to fluorescent cell mass localization, leading to a low background photopic response. For future studies, the number of injected cells may be increased to expand the graft area, which would eliminate the need to locate by fluorescence, as per Ribeiro et al., where the transplantation of 500,000 donor cells not only increased the graft area but also improved maturation compared with their previous studies using 150,000 cells (11). This would also potentially open up the possibility of additional functional testing, which was limited in this study not only because of the residual rod function in Cpfl1 but also the relatively small graft area. Regardless, using just 150,000 donor photoreceptors, we observed a 3- to 4-fold higher proportion of both ON- and OFF-responsive RGCs under mesopic and photopic conditions when comparing regions containing transplanted cells with nontransplant-containing regions. Of note, in previous experiments of transplantation into the Cpfl1 mouse, we were able to show a lack of photopic response by MEA in both sham and rod-only transplant controls (51). Together, this is a strong indicator that the increased response was due to light-evoked responses transmitted from the graft. While the ON RGC contribution of the graft versus endogenous rods cannot be resolved definitively, the introduction of L-AP4 isolated cone OFF bipolar responses. As cones are largely absent and completely dysfunctional in the Cpfl1 host, any cone-OFF bipolar response should be due to newly formed connections to the graft. Indeed, all ON responses were quenched by L-AP4, and OFF responses remained only in the transplanted region, giving strong evidence that there was photopic light–evoked signal transduction of transplanted cells through the cone-OFF pathway. This indicates that the well-matured and structurally incorporated photoreceptors in this study were functionally integrated and synaptically connected to the host retina.

In this study, we describe a human cone–specific reporter hiPSC line for the use of retinal organoid generation. Transplanted human cones and CRX^+^ photoreceptors extensively incorporated into a mouse model of cone degeneration. Incorporated grafts were well polarized and developed inner and outer segments. Further studies will be required to investigate details of the cellular and molecular requirements for structural incorporation and interaction with the host tissue allowing subsequent donor photoreceptor maturation. Such knowledge will be helpful to further optimize graft organization, outer segment formation, and synaptic connectivity, with the ultimate goal of improving visual perception. Nonetheless, the observed structural incorporation and subsequent in vivo polarization and maturation of the human photoreceptors, second-order neuron plasticity, and the lack of physical impediment to synaptic connectivity constitute encouraging evidence that transplanted human photoreceptors may have the potential to integrate into the remaining ONL of patients.

## Methods

### Vector production.

The piggyBac vector backbone PB-TRE-dCas9-VPR (52) was a gift from George Church (Wyss Institute at Harvard, Boston, Massachusetts, USA; Addgene plasmid 63800). All promoter elements and ORFs between the core insulator at the 5′ and the SV40 polA at the 3′ ends were removed using restriction enzymes and replaced with either a PCR-amplified rod or cone reporter cassette. PCR products were introduced into the piggyBac vector backbone using Gibson assembly cloning (53). For production of the cone reporter cassette, a PCR-amplified mCAR from LV-mCAR-eNpHR-EYFP (54) (a gift from Botond Roska, IOB, Basel, Switzerland) was assembled with an EGFP followed by a downstream WPRE-BGH-pA element. Finally, a PCR-amplified ubiquitin C promoter (UBC) blasticidin (Bla) cassette from the pLV-TRET-hNgn1-UBC-Bla vector (55) (a gift from Ron Weiss (MIT, Cambridge, Massachusetts, USA), Addgene plasmid 61473) was further added to both vector assemblies, resulting in the reporter plasmids PB-hRHO-DsRed-WPRE-BGH-pA-UBC-Bla and PB-mCAR-EGFP-WPRE-BGH-pA-UBC-Bla. The plasmid DNA was transformed in chemically competent bacteria (One Shot Stbl3, Thermo Fisher Scientific) following the manufacturer′s protocol. The correct sequences were confirmed with Sanger sequencing. While red fluorescent protein (RFP) was also introduced under the rhodopsin promoter, almost no RFP signal was detected even after 270 days in culture, however, for the purposes of a cone transplantation study, this was deemed irrelevant (data not shown).

### Generation of a hiPSC cone reporter cell line.

The Personal Genome Project hiPS cell line PGP1 (56) was a gift from George Church (https://www.encodeproject.org, accession number: ENCBS368AAA). The cells were cultured on Matrigel-coated wells (Corning, 354277) in mTeSR1 medium (STEMCELL Technologies, 85850) and passaged in the presence of ROCK Inhibitor InSolution Y-27632 (MilliporeSigma, 688001). The 4D-Nucleofector System (Lonza) was used to electroporate piggyBac and transposase vectors into PGP1 cells in suspension (X-Unit, P3 Primary Cell 4D-Nucleofector X Kit L, program CB-156) following the manufacturer’s protocol. After nucleofections, cells were selected with 20 μg/mL Bla (Thermo Fisher Scientific, A1113903) for 5 days. The selected cells were seeded at low densities and propagated until each single cell formed a colony. Colonies were selected and genotyped using primers specifically binding to rod and cone reporter cassettes. The monoclonal cell line carrying both reporter cassettes (PGP1dR) at passage numbers 33–38 was used for all further experiments. The primer sequences were as follows: *hRHO* forward, GGATACGGGGAAAAGGCCTCCACGGCCACTAGTAGTTAATGATTAACCCG; *hRHO* reverse, GACGTCCTCGGAGGAGGCCATGGTGGCTGCAGAATTCAGGGGATGACTCT; *mCAR* forward, CTGGGGGGATACGGGGAAAAGGCCTCCACGGCCACTAGTGGTTCTTCCCATTTTGGCTAC; *mCAR* reverse, GAACAGCTCCTCGCCCTTGCTCACCATGGTGGCTCTAGACCTCCAGCTCTGGTTGCTAAGCTGGC.

### hiPSC maintenance and differentiation of retinal organoids.

The mCar-GFP and Crx-mCherry iPSC lines (a gift from Olivier Goureau; ref. 22) were maintained in mTeSR1 (STEMCELL Technologies) on Matrigel-coated plates and split using ReleSR at room temperature (STEMCELL Technologies). Stem cells were differentiated into retinal organoids using a previously described optimized protocol (19) (see also Supplemental Methods).

### FACS of reporter^+^ cells.

Retinal organoids were dissociated in 20 U/mL papain, followed by gentle titration with a fire-polished glass pipette and further processing according to the manufacturer’s instructions (Papain Dissociation System, Worthington). The cell pellet was resuspended in MACS buffer (0.5% BSA, 2 mM EDTA in PBS) to a concentration of approximately 5 million cells/mL. The cell suspension was filtered through a 35 μm mesh and kept on ice for FACS. A FACSAria II or FACSAria III sorter was used to sort GFP^+^ and mCherry^+^ cells. Briefly, the forward-scatter (FSC-A) and side-scatter (SSC-A) areas were was used to discriminate cells from debris. Doublets were removed by gating the FSC area versus height and by the SSC height versus width. Dead cells were gated out using DAPI staining. Finally, GFP^+^ or mCherry^+^ cells were discriminated from autofluorescent cells using fluorescence detection at 505-525 nm (GFP) versus 579-594 nm (mCherry) or 636-677 nm (far red).

### Animals.

Adult Cpfl1-mutant mice (7–25 weeks of age) were used as recipients for cell transplantation (16). The Cpfl1 mouse colony maintained in the CRTD animal facility was founded from mice provided by Bernd Wissinger (Institute of Ophthalmic Research, Tübingen, Germany). Mice were maintained on a 12-hour light/12-hour dark cycle with ad libitum access to food and water.

### Transplantations.

Following FACS, GFP^+^ or mCherry^+^ cells were resuspended in MACS buffer (150,000 cells/μL) and injected into the subretinal space of host eyes as previously described (see also Supplemental Methods) (57). Directly following cell transplantation, 1 μL preservative-free triamcinolone acetonide suspension (80 μg/μL in NaCl prepared by the University Clinic Pharmacy, Dresden, Germany) was injected into the vitreous using a hand-held 10 μL Hamilton syringe with a blunt 34 gauge needle. Triamcinolone vitreal injections were repeated on a monthly basis.

### IHC.

Immunohistochemical analysis was performed as described previously (30) (see Supplemental Methods for details). For immunocytochemistry following dissociation and sorting of the retinal organoid cells, cells were resuspended in RM2 media, and laminin was added to each fraction. From each fraction, 50,000 cells were plated in flexiperm wells on a poly-D-lysine–coated (PDL-coated) slide. Cells were incubated at 37°C for 2 hours to allow attachment. Cells were then fixed for 15 minutes at room temperature, washed 3 times with PBS, and stained as described above for the frozen sections. Frozen sections and plated cells were mounted following antibody staining using AquaPolymount (Polysciences) and imaged using a Zeiss Apotome Imager Z2.

### Transmission electron microscopy and correlative light electron microscopy.

TEM of transplanted cones was performed as previously described (58, 59). Correlative light electron microscopy (CLEM) of immunolabeled sections was performed as described previously (60, 61) (see also the Supplemental Methods). Transmission electron microscopy (TEM) imaging was performed with a Jeol JEM 1400 and a transmission electron microscope (camera: Ruby, Jeol) running at 80 kV acceleration voltage.

### Isolation of transplanted cells for transcriptomic analysis.

Whole eye cups of transplanted eyes or organoids maintained in culture from the same differentiation round were dissociated with papain as described above (Papain Dissociation Kit, Worthington Biochemical Corp.; 20 U/mL). Cells were resuspended in MACS buffer and filtered through a 35 μm mesh before FACS and sequencing (method was modified based on ref. 62; see Supplemental Methods for details).

### Transcriptomic analysis.

Basic quality control of the resulting sequencing data was done using FastQC (version 0.11.6; https://www.bioinformatics.babraham.ac.uk/projects/fastqc/), and the degree of mouse contamination was assessed with FastQ Screen (version 0.9.3) (https://www.bioinformatics.babraham.ac.uk/projects/fastq_screen). Reads originating from mouse were removed with xengsort (version 2021-05-27; ref. 63). Reads were aligned to the human reference genome hg38 using the aligner gsnap (version 2020-12-16; ref. 64) with Ensembl 92 human splice sites as support. Uniquely mapped reads were compared on the basis of their overlap with the Ensembl 92 human gene annotations using featureCounts (version 2.0.1; ref. 65) to create a table of fragments per human gene and sample. Normalization of raw fragments based on library size and testing for differential expression between the different cell types and treatments were performed with the R package DESeq2 (version 1.30.1; ref. 66). Sample-to-sample Euclidean distance, Pearson’ and Spearman’s correlation coefficient, and principal component analysis (PCA) based upon the top 500 genes with the highest variance were computed to explore correlations between biological replicates and different libraries. To identify differentially expressed genes, counts were fitted to the negative binomial distribution, and genes were tested between conditions using the Wald test in DESeq2. Comparison of the GFP^+^ versus the GFP^–^ fractions included the age as a covariate, while all other comparisons just included the specific groups. The resulting *P* values were corrected for multiple testing with the Independent Hypothesis Weighting package (IHW, version 1.18.0) (67, 68). Genes with a maximum FDR of 5% (adjusted *P* ≤ 0.05) were considered significantly differentially expressed.

Panther was used for gene enrichment analysis (69). Differentially expressed genes from our data set were run through the statistical overrepresentation test function using the whole human genome as a reference list. Fisher’s exact test with a calculated FDR was selected, and output was condensed by hierarchical clustering of GO terms to reduce repetitive pathway findings. Morpheus (https://software.broadinstitute.org/morpheus) was used to create heatmaps.

Raw data and processed counts were deposited in the NCBI’s Gene Expression Omnibus (GEO) database (GEO GSE201219).

### Electrophysiological recordings with MEA.

A Glass MEA with 256 electrodes of 30 μm diameter and a spacing of 200 μm spanning an area of 3 mm × 3 mm (256MEA100/30iR-ITO) with a recording headstage (MEA256-System, Multi Channel Systems [MCS]) below the microscope was used for all experiments.

The preparation of ex vivo retinas was performed in carbonated (95% O_2_, 5% CO_2_) Ames’ medium (Ames A 1420, MilliporeSigma plus NaHCO_3_). Following euthanasia of the mouse, the eyes were opened via a small needle incision above the ora serrata. After removal of the lens, the eye was cut in half and the graft located with a stereomicroscope (Leica M80), equipped with a fluorescence illumination unit. The retina with the graft was then separated from the sclera and RPE and trimmed with a scalpel, and the vitreous was removed. The retina was placed ganglion cell–side up on a filter paper and transferred RGC-side down onto the coated recording electrodes (Cell-Tak, Corning), as described in detail in a previous report (70). The filter paper was then removed. The other half of the retina was prepared in the same way as for the nontransplant control.

A patterned light stimulus created by an organic light-emitting diode (OLED) display (DSVGA monochrome green XLT, eMagin), in combination with GEARS software (71), was used, allowing for bin,ary checkerboard white noise (bwn) stimulation. The OLED was coupled to the microscope with an adapter and its light was projected onto the sample through a ×2.5 objective. The OLED’s power was derived as *P =* 0.7 μW for full-field illumination, which can be calculated into photoisomerizations equaling approximately 1 × 10^5^ R*/photoreceptor/s for both rods and medium-wavelength cones. We generated pseudorandom, binary (green and black) checkerboard stimuli, where, at every stimulus frame, the intensity of each checker was drawn from a binary distribution, with a temporal frequency of 38 Hz (frame duration of 26 ms), a total duration of 25 minutes, and a resolution of 30 × 30 pixels, resulting in an illuminated area of 3.2 × 4.2 mm.

During RGC activity recording, the MEA chamber was continuously perfused with Ames’ solution at a rate of 2–4 mL/min. The temperature of the MEA chamber was maintained at approximately 36°C by heating the bottom of the recording chamber and the perfusion inlet. To ensure that RGC-OFF responses were driven by injected photoreceptors, the experiments were performed before and after addition of the mGluR6 blocker L-AP4 (50 μM, Tocris, catalog 0103). Extracellular voltages were recorded using the software MC_Rack (MCS) and preprocessed using a second-order Butterworth highpass filter (300 Hz), before spike detection (see Supplemental Methods for details).

### Image processing.

Images and graphs were processed and generated using ImageJ (NIH), Zen Blue Software (Zeiss), Affinity Designer (Serif), and GraphPad Prism 7 (GraphPad Software).

RCVRN and cone arrestin quantification by immunocytochemistry was performed using CellProfiler, version 3.1.9. The total graft area was quantified using the ZEN Blue Image Analysis Wizard of Axioscan images of every fourth serial retinal section throughout the whole eyecup of each sample, and then each individual GFP^+^ cell cluster with an associated area was analyzed for its level of incorporation. On the basis of the host ONL apical border, a line was drawn and the graft area below and above quantified. The groups were defined as follows: 5%–20% of the area below the apical border, starting to incorporate; 20%–80% below, partially incorporated; greater than 80% below, fully incorporated. Inner segment outgrowths were counted manually. For comparison of inner segment formation on the basis of interaction with the host retina, the graft area and number of inner segments were measured ±50 μm of the retinal contact sites in D250 plus 10-week samples.

### Statistics.

Statistical significance was calculated using a 1-way ANOVA with Tukey’s multiple-comparison test. A *P* value of less than 0.05 was considered statistically significant. For a detailed statistical analysis of the transcriptional data and spike sorting, see the respective sections.

### Study approval.

All animal experiments were approved by the ethics committee of the Technische Universität Dresden and the Landesdirektion Dresden (approval numbers: TVV 16/2016 and TVV 38/2019). All relevant European Union regulations, German laws (Tierschutzgesetz), the Association for Research in Vision and Ophthalmology (ARVO) statement on the Use of Animals in Ophthalmic and Vision Research, and the NIH *Guide for the Care and Use of Laboratory Animals* (National Academies Press, 2011) were strictly followed for all animal work.

## Author contributions

SJG, KT, and MA conceived the study. SJG, KT, MR, MC, OB, SW, AS, MZ, MV, TK, OG, MOK, VB, GZ, and MA designed and/or performed the experiments. SG, KT, and MA wrote the manuscript, with input from all authors. The order of co–first authors’ names was assigned randomly.

## Supplementary Material

Supplemental data

## Version 1 04/28/2022

In-Press Preview

## Version 2 06/15/2022

Electronic publication
